# Is There Any Effect of Change in Pre-Wash and Post-Wash Semen Parameters on the Success of Intrauterine Insemination?

**DOI:** 10.3390/jpm14010043

**Published:** 2023-12-28

**Authors:** Ali Yavuzcan, Engin Yurtçu, Betül Keyif, Şeyma Osmanlıoğlu

**Affiliations:** 1 Department of Obstetrics and Gynecology, Sağlık Bilimleri University, Ankara 06010, Türkiye; 2 Department of Obstetrics and Gynecology, Düzce University, Düzce 81620, Türkiye; enginyurtcu@duzce.edu.tr (E.Y.);; 3 Department of Obstetrics and Gynecology, Ankara Medipol University, Ankara 06050, Türkiye

**Keywords:** intrauterine insemination, post-wash, pre-wash, semen parameters, success

## Abstract

(1) Background: We aimed to investigate the effect of change in pre-wash and post-wash semen parameters on intrauterine insemination (IUI) success in a homogenous study group. (2) Methods: IUI cycles conducted at an infertility clinic were included in this study. Patient records were examined retrospectively. Δ sperm count (per mL) was calculated as [pre-wash sperm count (per mL)–post-wash sperm count (per mL)]. Δ Total progressive motile sperm count (TPMSC) was also calculated as (post-wash TPMSC-pre-wash TPMSC). (3) Results: No statistically significant difference was detected in terms of Δ sperm count (*p* = 0.38), and Δ TPMSC (*p* = 0.76) regarding the clinical pregnancy rate (CPR). There was no statistically significant difference between CPR (+) and CPR (−) groups in terms of post-wash sperm count, TPMSC, TPMSC ≥ 10 × 10⁶, TPMSC ≥ 5 × 10⁶ (*p* = 0.65, *p* = 0.79, *p* = 0.49, *p* = 0.49, respectively). The live birth rate (LBR) showed no statistically significant differences except for a pre-wash TPMSC ≥ 10 × 10⁶ (*p* = 0.02). Through the performed ROC analysis, no statistically significant cutoff value could be set for the pre-wash TPMSC. (4) Conclusions: There is only a pre-wash TPMSC ≥ 10 × 10⁶ that showed a significant role in the success of IUI, even when considering all other pre-wash and post-wash semen parameters. Δ sperm count and Δ are not useful markers for IUI success.

## 1. Introduction

Infertility is a common health problem. One out of every four couples in developing countries is affected by infertility throughout their lives [1]. It is reported that approximately 30% of infertility factors are related to men, about 40% to both men and women, and 20–70% to women [2,3]. Therefore, women play a more significant role in infertility. Treatment options for infertile men are limited. Traditional infertility treatments primarily focus on women and include hormone therapy, tubal plastic surgery, and assisted reproductive technologies [4]. One of the treatment alternatives for infertile men is intrauterine insemination (IUI). IUI is a common option for couples facing mild male factor issues [5]. The logic of IUI is to achieve high gamete density within the tubal area. IUI is also indicated in cases of unexplained infertility, anovulation, and endometriosis associated with healthy fallopian tubes or mild endometriosis. Typically, these couples undergo three inseminations before in vitro fertilization (IVF) treatment [6,7,8]. Intrauterine insemination (IUI) is a more patient-friendly, easily applicable, and cost-effective treatment method compared to in vitro fertilization (IVF). In IUI treatment, there is no need for daily high-dose parenteral gonadotropin administration, close monitoring, frequent hormone monitoring during treatment, or expensive embryology laboratories as in IVF. Complications such as ovarian hyperstimulation syndrome and multiple pregnancies, which may arise during IVF treatment, are very rare in IUI.

It is known that some variables associated with female and male partners predict the success of IUI [9]. These variables are the type and etiology of infertility, the number of IUI cycles, the count of dominant follicles before human chorionic gonadotropin (HCG) administration, and sperm parameters in the pre-wash sperm analysis and post-wash sperm analysis [10,11]. Although specific cutoff levels for semen parameters predicting pregnancy achievement have been reported, their sensitivity in predicting successful pregnancy is low; however, they show a better ability to predict failure to conceive [12]. Patients with a total sperm count (TSC) < 5 × 10^6^ in the natural sperm analysis are considered to have severe oligospermia [10], and they are usually offered intracytoplasmic sperm injection (ICSI) or conventional IVF. A recent review suggested that for couples with a post-wash total progressive motile sperm count (TPMSC) > 1 million, regardless of sperm morphology, IUI is the most cost-effective treatment before initiating IVF [12]. Furthermore, couples with a post-wash TPMSC of more than 5 million demonstrated a high pregnancy rate [13], while a strong negative correlation was reported between post-wash low TPMSC (<5 million) and pregnancy [14].

The recent guideline from the American Urological Association (AUA) and the American Society for Reproductive Medicine (ASRM) emphasizes that a semen analysis is a fundamental test in evaluating infertile couples [14]. If one or more abnormal parameters are detected in the initial semen analysis, a retest is recommended within 2 to 4 weeks. Evaluating post-wash semen results, alongside the initial semen analysis, is necessary not only in men with oligospermia but in all men. Decisions regarding IUI patient selection [13] and the choice between ICSI and IVF in infertile men are based on pre-wash and post-wash sperm parameters [15].

Fundamentally, three primary methods have been identified for sperm washing. Spermatozoa selection based on swimming ability, known as the “swim-up technique”, involves overlaying liquefied semen with a culture medium. Another method, “density gradients”, separates motile, morphologically normal sperm using a high-gradient-concentration solution after pipetting the semen sample onto a density column and centrifuging it. The third method, “conventional washing”, dilutes the semen sample with a medium, followed by centrifugation. Subsequently, the pellet is re-suspended in a small amount of medium [16,17]. There is currently insufficient evidence to advise any specific technique for semen preparation [17]. The swim-up technique, apart from simple washing methods, is the oldest and most utilized sperm preparation method. It is still widely employed in IUI and IVF laboratories worldwide. The density gradient technique is more practical than the swim-up technique. It is easier to standardize and yields more consistent results [17].

Semen-washing procedures eliminate immotile sperm cells, prostaglandins, immature germ cells, leukocytes, antigenic proteins, and infectious agents from the environment. Consequently, the formation of free oxygen radicals may decrease after sperm preparation, potentially enhancing sperm quality. Post-wash semen parameters may not always correlate with pre-wash semen parameters [12]. The sperm preparation process aims to improve semen quality by generating a preparation with an increased number of progressively motile cells [15]. Sperm count and motility can change significantly before and after semen processing, but data on the effect of this change in semen processing on sperm analysis measurements for IUI are limited [8,18].

Following washing, a decrease in sperm concentration in milliliters (mL) is usually detected. TPMSC, however, increases in most patients after washing. We formulated these changes as Δ sperm count [pre-wash sperm count (per mL)**—**post-wash sperm count (per mL)] and Δ TPMSC (post-wash TPMSC**—**pre-wash TPMSC). We aimed to explore the influence of changes in pre-wash and post-wash semen parameters on IUI success within a homogeneous study group.

## 2. Material and Methods

### 2.1. Study Population

Two hundred and seventy-six IUI cycles were conducted at Düzce University Health and Training Center Infertility Clinic between January 2020 and December 2020. This study received approval from the ethics committee at the Düzce University Medical Faculty in Düzce (Approval No.: 2023/151). Patient records were examined retrospectively. All patient-related data had been documented. Our study was conducted based on the principles of the Helsinki Declaration. Caucasian women aged 18–38 years, with one or two tubal passages (confirmed through diagnostic laparoscopy or hysterosalpingography), and a follicle-stimulating hormone (FSH) value below 12 pg/mL on the 3rd day of menstruation, were all included in our study. Females were classified into three age groups: the early reproductive period (<25 years old), the mid-reproductive period (25–34 years old), and the late reproductive period (>35 years old), based on their respective reproductive stages [19]. Similarly, men were categorized into the age groups of <35 years, 35–39 years, and ≥40 years [20].

### 2.2. Ovarian Stimulation and IUI Protocol

**Figure 1:**
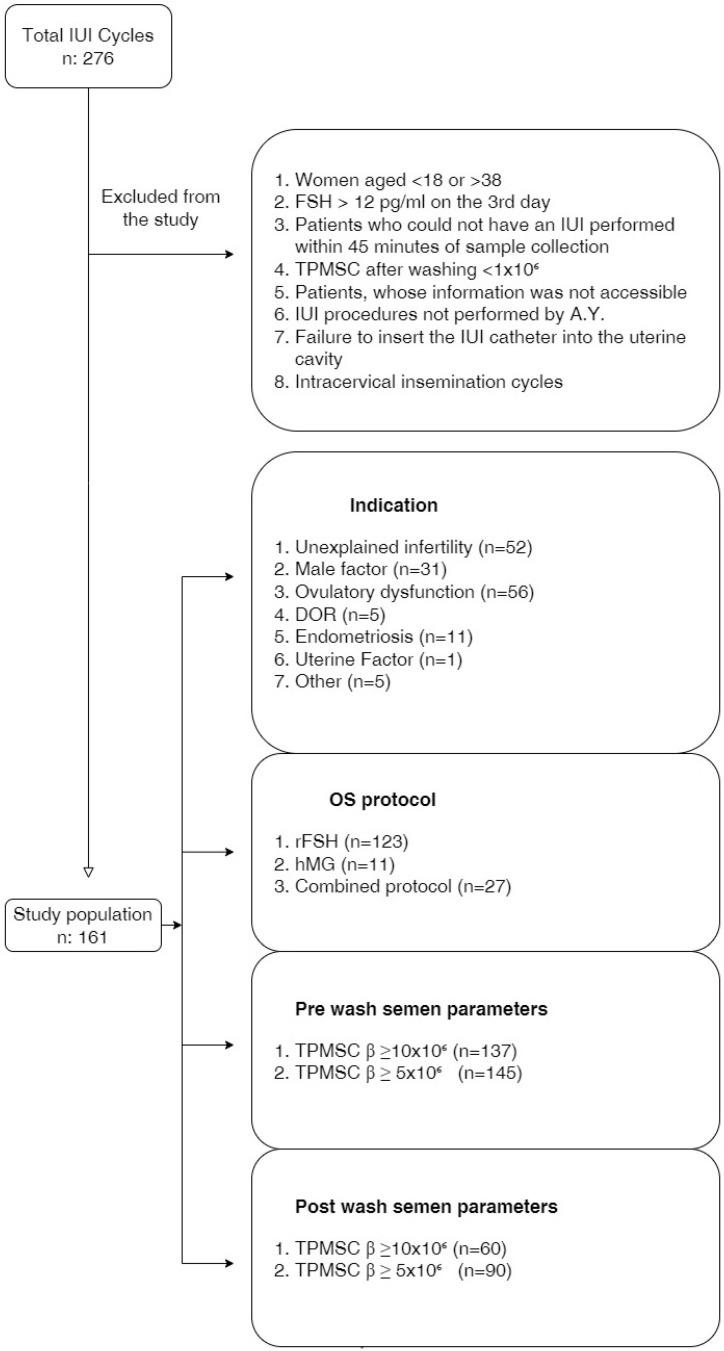
Flowchart of study population.

Ovulation induction (OI) was started in patients with endometrial thickness < 4 mm and without follicles > 10 mm by performing a vaginal ultrasonographic examination on the 2nd or 3rd day of the cycle. OI was conducted using one of three randomly selected regimens. Combined protocol: after 5 mg/day of oral letrozole for 5 days, 75 IU parenteral human menopausal gonadotropin (HMG) or recombinant FSH (rec-FSH) was administered on the 6th and 7th days of induction. In the other two protocols, on the 2nd or 3rd day of the menstrual cycle, rec-FSH or HMG 75 units were given for 7 days. Patients were called for control on the 8th day after 7 days of OI. When the transvaginal measured dominant follicle reached ~18 mm in size, ovulation was triggered with the parenteral HCG injection. A total of 34–36 h after triggering ovulation with hCG, IUI was applied with the sperm sample that was washed in the andrology laboratory. After OI, patients in whom no dominant follicle was detected in the control were called for another visit after 3 days of increasing the amount of parenteral gonadotropin by 50%. The cycle was canceled for patients who had no follicle development at the end of 21 days of induction. Additionally, the IUI procedure was canceled in patients with ≥3 follicles with a diameter of 16 mm. Patients who could not have an IUI performed within 45 min of sample collection, TPMSC after washing <1 × 10^6^, patients with a low ovarian reserve, patients whose information was not accessible, IUI procedures not performed by A.Y., failure to insert the IUI catheter into the uterine cavity, or intracervical insemination cycles were also excluded. Included and excluded patients are summarized in the study flowchart (Figure 1).

### 2.3. Semen Analysis

After the initial semen analysis, the sample underwent processing by placing 4 mL of raw semen into a differential density gradient column, composed of 1 mL of 40% pure sperm and 1 mL of 80% pure sperm, followed by centrifugation at 350× *g* for 20 min. Subsequently, the 40% layer and seminal plasma fraction were extracted from the test tube, leaving the undisturbed 80% layer [18]. Around 6–8 mL of a sperm washing medium containing 5% HSA and bicarbonate concentration was added to the 80% layer. This solution underwent centrifugation at 550 g for 10 min. The sperm pellet was subsequently diluted to approximately 0.5 mL The semen analysis was conducted following the guidelines of the World Health Organization (WHO) from the year 2010. Δ sperm count (per mL) was calculated as [pre-wash sperm count (per mL)—post-wash sperm count (per mL)]. Additionally, Δ TPMSC (post-wash TPMSC—pre-wash TPMSC) was calculated.

### 2.4. Insemination

The IUI procedure was performed using a flexible plastic catheter in a patient positioned in the dorsal lithotomy position [18]. After the completion of the IUI procedure, the patient was advised to remain in a supine position for at least 15 min. After the IUI procedure, starting from the day after, 200 mg of micronized natural progesterone was administered vaginally for 14 days. The patient was called in for a serum ßHCG test on the 14th day after IUI.

### 2.5. Patient Demographics and Outcome Assessment

Age, infertility indication, ovarian reserve assessment at day 3 of the menstrual cycle, previous pregnancies, and births were used as patient demographics. The number of IUI cycles and drugs used for ovarian induction were used as cycle characteristics. Abortus, ovarian hyperstimulation syndrome (OHSS), multiple gestations as a result of IUI, and the presence of teratozoospermia were also assessed. The first outcome measure was the live birth rate (LBR), which was defined as live birth delivery past 24 weeks of gestation [18]. The clinical pregnancy rate (CPR), which was our second main outcome, was defined through documentation of the fetal heartbeat with ultrasound [18]. ßHCG positivity was defined as a positive quantitative serum ßHCG test.

### 2.6. Statistical Analyses

The data were analyzed using IBM SPSS 21.0 software (SPSS Inc., Chicago, IL, USA). Results were presented as the median [min–max] or mean ± SD depending on the overall distribution of variables. The normality of distribution was determined using the Shapiro–Wilk test. According to the results, non-parametric tests were preferred. Continuous variables were compared using the Mann–Whitney U test and the categorical variables were compared using the Chi-square test or the Fisher’s exact test, where appropriate. A *p*-value < 0.05 was considered statistically significant.

## 3. Results

### 3.1. Demographic Data

The average age of women was 28.15 ± 4.1 years, and for men, it was 32 ± 5.2 years. The most common indications observed were ovulatory dysfunction at 34.8% (n = 56) and unexplained infertility at 32.3% (n = 52). CPR and LBR were 14.3% (n = 23) and 10.6% (n = 17), respectively. Of the patients, 93.2% (n = 150) were diagnosed with primary infertility, while 6.8% (n = 11) had secondary infertility. Table 1 summarizes the demographic data for the study population including the patient’s age, serum FSH, LH, estradiol, prolactin, TSH, gravidy, and parity.

### 3.2. Comparison of the Pre-Wash and Post-Wash Semen Parameters Regarding the CPR

A comparison of the pre-wash and post-wash semen parameters regarding the CPR showed no statistically significant differences (Table 2). Additionally, no statistically significant differences were detected in terms of Δ sperm count per mL (*p* = 0.38), and Δ TPMSC (*p* = 0.76) regarding the CPR (Table 2).

### 3.3. Comparison of the Pre-Wash and Post-Wash Semen Parameters Regarding the LBR

LBR showed no statistically significant differences except for a pre-wash TPMSC ≥ 10 × 10⁶ (*p* = 0.02) (Table 3). However, despite performing an ROC analysis, no statistically significant cutoff values could be established for the pre-wash TPMSC.

### 3.4. Comparison of the Reproductive Phases of Couples and Ovarian Stimulation Protocols in Terms of CPR and LBR

The average age of the women showed no statistically significant differences in terms of CPR (*p* = 0.18) and LBR (*p* = 0.10). Similarly, the age of the men did not show any significant differences in terms of CPR (*p* = 0.16) and LBR (*p* = 0.19). The comparison of age categories, which shows the reproductive phases, also showed no statistical differences regarding the CPR, and LBR for both women (*p* = 0.79 and *p* = 0.90, respectively) and men (*p* = 0.06 and *p* = 0.21, respectively). The rec FSH group had the highest CPR (n = 14, 11.8%). A comparison of stimulation protocols showed a borderline significant difference regarding CPR (*p* = 0.05). There was not any statistically significant difference in terms of the stimulation protocol for the LBR (*p* = 0.08).

## 4. Discussion

We hypothesized that the reported different values in studies regarding the pre-wash or post-wash minimum sperm count and total motile sperm count necessary for CPR and LBR were due to population variability. Therefore, we examined two new semen parameters that we believed would correlate with CPR and LBR rates in IUI cycles, even when used in different populations. We anticipated that semen processing procedures would enhance semen quality by producing a preparation containing a high concentration of progressive motile, healthy, and high-quality cells. We estimated that a decrease in sperm concentration per mL after semen processing might reduce the likelihood of sperm reaching the fallopian tube and encountering the oocyte, thereby potentially affecting the overall success rate of IUI. The sperm processing procedure eliminates unnecessary and potentially harmful components present in the ejaculate, while the insemination process concentrates motile sperm into a relatively small volume, which is then deposited into the intrauterine environment [21]. However, our findings indicated that the Δ sperm count per mL did not show a statistically significant difference in CPR (+) and LBR (+) IUI cycles compared to controls. Progressive motility of sperm is essential for reaching and fertilizing the oocyte within the fallopian tube. We anticipated that CPR and LBR would increase in patients who showed a greater increase in TPMSC in semen after washing. Nevertheless, we found no statistically significant differences between the CPR (+) and CPR (−) groups, as well as the LBR (+) and LBR (−) groups, in terms of Δ TPMSC.

IUI has become a widely utilized technique for couples experiencing fertility issues. One of the critical components of the success of IUI is the quality of sperm used for insemination [7]. Spermiogram parameters play a crucial role in assessing couples’ suitability for an IUI cycle, continuously attracting research attention and clinical interest. However, previous reports indicated that no universally accepted parameter from pre-wash or post-wash semen analyses reliably predicts the outcome of IUI treatment [22,23,24,25]. In our study, patients with a post-wash TPMSC of <1 × 10^6^ were not included. Additionally, IUI was not performed on women ≥38 years old, and the IUI procedure was carried out by the same clinician. For sperm preparation and washing, we employed the conventional sperm washing method. In the Cochrane review updated in 2019, the efficacy of specific semen preparation techniques in increasing pregnancy rates among subfertile couples undergoing insemination remains uncertain [17]. In addition, a recent study conducted in 2022 suggests that the type of sperm preparation method does not affect pregnancy rates in IUI cycles [26]; in our study, all semen-washing procedures were consistently conducted in the same laboratory by the same team of technicians.

Data supporting the utilization of semen parameters are divided among different populations [24]. Within the infertile population, there is no consensus regarding pre-wash and post-wash semen parameters for recommending IUI or IVF. Strategic identification of couples with a potential likelihood of achieving pregnancy through IUI can decrease the rate of direct transition to IVF and ICSI treatments for the infertile population. Currently, there exists no globally standardized cutoff value defined for semen parameters that predicts the success of IUI treatment. Luco et al. claimed that post-processing semen analysis results do not predict pregnancy more effectively than pre-processing semen analysis results. They emphasized that the total motile sperm count (TMSC) is not a predictive parameter for pregnancy [18]. In a 2022 study, Mathes et al. were unable to establish a pre-wash TMSC threshold for recommending IUI, but they explained a positive correlation between TMSC and IUI success [22]. A large-scale study suggested that a pre-wash TMSC cutoff value of 2 million is indicative of pregnancy and LBR [27]. According to another recent study, acceptable pregnancy rates can be achieved with post-processing TPMSC in the range of 3–10 million with IUI [28]. However, this study emphasized that both pre-wash and post-wash TPMSC possess weak predictive value for post-IUI CPR [28]. In our study, we found a higher cutoff value for TPMSC compared to previous studies. Our study found that a pre-wash TPMSC ≥ 10 × 10^6^ is the only pivotal factor influencing the LBR after IUI (*p* = 0.02). Despite performing the ROC analysis, we were unable to determine a significant cutoff value for pre-wash TPMSC.

Ombelet et al. proposed threshold semen parameters for the application of IUI. They emphasized that a normal morphology > 4 in a pre-wash semen analysis could serve as a predictor for pregnancy with low sensitivity [7]. Mathes et al. claimed that men with a morphology > 4% exhibited higher clinical pregnancy rates (OR: 0.84) [22]. Conversely, DeVilbiss et al. investigated 2369 couples seeking fertility consultations at four centers. They reported that low morphology was only related to IVF success, not IUI [24]. Stanhiser et al. found no differences in CPR following IUI among infertile couples with normal and abnormal sperm morphology, including severe teratospermia [29]. Similarly, Geraldo Orrego et al. reported that isolated teratozoospermia was not a contraindication for IUI and there was no defined cutoff point for sperm morphology predicting pregnancy [30]. A recent review corroborated these findings, indicating an absence of consistent effects of sperm morphology on CPR [31]. In the presence of teratozoospermia, the ideal treatment approach remains unclear. However, like many previous studies, our research also indicated that the presence of teratozoospermia did not significantly impact the success rates of IUI concerning both CPR (*p* = 0.344) and LBR (*p* = 0.14).

There is an age-related decline in sperm motility and concentration [32]. Huniadi et al. underscored the importance of male age as a decisive factor influencing the outcome of IUI [33]. Starosta et al. reported that advanced paternal age negatively impacts pregnancy rates in cycles undergoing IUI [34]. However, in our study, we found that male age did not have a significant effect on IUI success. Notably, we have only one man > 45 years old in our study, whereas studies have indicated a dramatic decline in sperm motility and concentration beyond this age threshold [32]. The most crucial factor affecting a woman’s likelihood of conceiving and the outcome of infertility treatments is her age. Several studies have reported a significant decrease in pregnancy rates among infertile elderly women. Consequently, it has been suggested that IUI should not be performed in women older than 38–40 years due to this decline in success rates [33,34,35]. Osaikhuwuomwan et al. also claimed that the pregnancy rate was highest in the younger age group who were below 30 years [36]. On the other hand, Madbouly and colleagues found that female age did not affect IUI success [37]. In our study, female age did not demonstrate a statistically significant difference concerning CPR (*p* = 0.18) or LBR (*p* = 0.10). We classified women into three age groups according to the reproductive period and did not find any statistical difference regarding the CPR, and LBR (*p* = 0.79 and *p* = 0.90, respectively). We excluded patients ≥ 38 years in this study and our results did not differ between age groups in this homogenous study population. Wang et al. similarly classified IUI patients into three distinct reproductive phases as in our study and identified significant differences in CPR among these phases [38]. Data comparing the relationship between the success of IUI and women’s reproductive phases are limited.

The current recommendation suggests the incorporation of post-wash sperm parameter assessment in routine male infertility evaluations to aid in appropriate patient counseling and selection of suitable assisted reproductive techniques [37]. However, Cohlen et al. declared in their review that it is not possible to clearly define the lower threshold levels of pre- or post-wash sperm parameters required for the implementation of IUI [39]. Numerous sperm parameters, both pre- and post-washing, have been identified to preselect suitable patients for IUI treatment. Nevertheless, the predictive values of these parameters vary widely, with a higher predictive power for non-conception. Evidence suggests that having a sperm morphology rate of <4% does not necessarily preclude IUI for the patient. Currently, the available guidelines on this issue are limited to those jointly issued by the AUA and ASRM [14]. However, this guideline only states that negative effects on pregnancy rates are observed in patients with post-wash TPMSC < 5 × 10^6^. In our study, we aimed to pinpoint a consistent sperm parameter significantly influencing the success of IUI by leveraging both commonly used sperm parameters from previous studies and two newly devised parameters. Nonetheless, our study has some limitations. It is important to note that the retrospective nature of our analysis limits our ability to establish causality. Moreover, we would have preferred a larger number of patients to be included in the study. Future research should prioritize prospective studies with larger sample sizes, potentially involving multicenter studies with participants from diverse populations. Such studies can better explore globally accepted semen parameters to effectively identify suitable candidates for successful IUI treatments.

## 5. Conclusions

Our findings highlight the significant role of pre-wash TPMSC ≥ 10 × 10^6^, even when considering all other pre-wash and post-wash semen parameters, in the success of IUI. Notably, Δ sperm count and Δ TPMSC do not demonstrate utility as reliable markers for predicting IUI success.

## Figures and Tables

**Table 1:** Demographics.

	n (%)	Mean ± SD
Mean age of women (years)	161	28.15 ± 4.1
FSH levels (mIU/mL)	161	6.2 ± 1.96
LH levels (mIU/mL)	161	8.9 ± 6.87
Estradiol levels (pg/mL)	160	63.4 ± 61.72
Prolactin levels (ng/mL)	159	18.1 ± 8.2
TSH levels (mIU/mL)	161	2.2 ± 1.1
Mean age of men (years)	161	32 ± 5.2
Number of cycles	161	1.3 ± 0.49
Gravida	161	0.2 ± 0.5
Parity	161	0.1 ± 0.3
Infertility type		
Primary	150 (93.2)	
Secondary	11 (6.8)	
Indication		
Unexplained infertility	52 (32.3)	
Male factor	31 (19.3)	
Ovulatory dysfunction	56 (34.8)	
DOR	5 (3.1)	
Endometriosis	11 (6.8)	
Uterine factor	1 (0.6)	
Other	5 (3.1)	
OS protocol		
rFSH	123 (76.4)	
hMG	11 (6.8)	
Combined protocol	27 (16.8)	
Clinical pregnancy rate	24 (15)	
Live birth rate	23 (14.3)	
Abortus	17 (10.6)	
Multiple gestation	9 (5.6)	
OHSS	0	
Presence of teratozoospermia	24 (15)	

DOR: Diminished Ovarian Reserve; FSH: Follicle-Stimulating Hormone; hMG: Human Menopausal Gonadotropin; LH: Luteinizing Hormone; OS: Ovarian Stimulation; OHSS: Ovarian Hyperstimulation Syndrome; rFSH: Recombinant FSH.

**Table 2:** Semen parameters and CPR.

	CPR (+) Mean/n (%)	CPR (−) Mean/n (%)	*p*-Value
Pre-wash			
Sperm count (per mL) ^α^	71.24	82.63	* 0.28
TPMSC ^α^	75.74	81.88	* 0.56
TPMSC ^β^ ≥ 10 × 10⁶	17 (12.4)	120 (87.6)	** 0.18
TPMSC ^β^ ≥ 5 × 10⁶	22 (14.2)	133 (85.8)	** 1.0
Percentage of normal morphology	77.39	81.6	* 0.68
Teratozoospermia ^β^			
(+)	5 (21.7)	19 (13.8)	** 0.344
(−)	18 (78.3)	119 (86.2)	
Post-wash			
Sperm count (per mL) ^α^	76.89	81.68	* 0.65
TPMSC ^α^	78.58	81.4	* 0.79
TPMSC ^β^ ≥ 10 × 10⁶	7 (11.7)	53 (88.3)	*** 0.49
TPMSC ^β^ ≥ 5 × 10⁶	16 (16)	84 (84)	*** 0.49
Delta values			
Δ sperm count (per mL) ^α^	73.02	82.33	* 0.38
Δ TPMSC ^α^	76.48	81.75	* 0.61

*p*-Value < 0.05 was considered statistically significant. *** Pearson Chi-Square; ** Fisher’s Exact Test; * Mann–Whitney Test. ^α^: mean; ^β^: n (%); CPR: clinical pregnancy rate; TPMSC: total progressive motile sperm count.

**Table 3:** Semen parameters and LBR.

	LBR (+) Mean/n (%)	LBR (−) Mean/n (%)	*p*-Value
Pre-wash			
Sperm count (per mL) ^α^	64.74	82.92	* 0.28
TPMSC ^α^	66.15	82.75	* 0.56
TPMSC ^β^ ≥ 10 × 10⁶	11 (8.03)	126 (91.97)	** 0.02
TPMSC ^β^ ≥ 5 × 10⁶	16 (10.3)	139 (89.7)	*** 0.64
Percentage of normal morphology	17 (10.6)	144 (89.4)	* 0.69
Teratozoospermia ^β^			
(+)	5 (29.4)	19 (13.2)	** 0.14
(−)	12 (70.6)	125 (86.8)	
Post-wash			
Sperm count (per mL) α	74.21	81.8	* 0.65
TPMSC α	75.44	81.66	* 0.79
TPMSC ^β^ ≥ 10 × 10⁶	55 (91.67)	5 (8.33)	*** 0.6
TPMSC ^β^ ≥ 5 × 10⁶	11 (11)	89 (89)	*** 1
Delta values			
Δ sperm count (per mL) ^α^	65.97	82.77	* 0.38
Δ TPMSC ^α^	65.82	82.79	* 0.16

*p*-Value < 0.05 was considered statistically significant. *** Pearson Chi-Square; ** Fisher’s Exact Test; * Mann–Whitney Test; ^α^: Mean; ^β^: n (%); LBR: Live Birth Rate; TPMSC: Total Progressive Motile Sperm Count.

## Data Availability

Data will be available on request.
